# Translating Traditional Herbal Use into Medication-Safety Decisions: A Proposed Community-Contextualized Translational Ethnopharmacy Framework for Pharmacy Practice

**DOI:** 10.3390/pharmacy14070141

**Published:** 2026-09-23

**Authors:** Ziad Dabour, David H. Kinder

**Affiliations:** 1 Department of Medical Laboratory Sciences, Zefat Academic College, Safed 1320611, Israel; 2 Department of Pharmaceutical and Biomedical Sciences, College of Pharmacy, Ohio Northern University, Ada, OH 45810, USA

**Keywords:** clinical pharmacy, community pharmacy, medication reconciliation, medication safety, herb–drug interactions, traditional medicine, ethnopharmacy, pharmacovigilance, evidence-to-decision

## Abstract

Traditional herbal preparations are frequently used alongside prescription and non-prescription medicines, yet conventional medication reconciliation may not preserve the preparation details needed to interpret interaction and safety evidence. We propose the community-contextualized translational ethnopharmacy framework (CTEF) as a testable conceptual architecture for pharmacy practice. Developed through a problem-oriented conceptual synthesis and a targeted, non-systematic comparator mapping, CTEF treats the exact reported preparation as the clinical exposure and embeds it within a review of the patient’s complete medication and clinical context. The revised framework is iterative and comprises five functions: exposure characterization; comprehensive medication and clinical reconciliation; appraisal of the evidence profile and transferability to the reported preparation; consequence-sensitive action selection using patient vulnerability and explicit escalation/de-escalation rules; and culturally responsive communication, documentation, and follow-up. The central hypothesis is that, compared with a prespecified standard medication-reconciliation workflow, CTEF will improve exposure-characterization completeness, inter-rater reproducibility, and concordance with blinded external adjudication without unacceptable increases in consultation burden, unnecessary escalation, or patient-reported communication harm. CTEF is not a scale, treatment guideline, or validated clinical decision rule. Staged co-design, simulation, reliability, feasibility, comparative, and cross-cultural studies are required before clinical adoption.

## 1. Introduction

### 1.1. Traditional Herbal Use as a Medication-Safety Problem

Traditional herbal preparations are commonly used alongside prescription medicines, over-the-counter (OTC) products, and dietary supplements [1]. Older adults and people with multimorbidity are particularly exposed to interaction risk because polypharmacy is common and because herbal use is frequently undisclosed [2,3]. Community-pharmacy studies further show inconsistent questioning, documentation, counseling, and adverse effect reporting for complementary and herbal medicines [4,5,6,7,8]. If a traditional preparation is not elicited, it cannot be reconciled, evaluated, or monitored.

The medication-safety problem is not captured adequately by the plant name alone. The clinically relevant exposure may depend on botanical identity, plant part or formulation, extraction medium, concentration, amount, route, timing, duration, source, and co-administered medicines. Existing interaction screening tools are valuable but can provide incomplete or inconsistent herbal coverage [9], and evidence generated from a standardized extract may not transfer directly to an incompletely characterized household preparation. Conversely, limited mechanistic evidence should not automatically trigger alarm when the patient’s vulnerability and the clinical consequence of a missed interaction are low.

### 1.2. Scope, Definitions, and the Target Decision

In this article, a traditional herbal preparation means a medicinally intended botanical exposure reported by a patient, whether homemade or commercially supplied, and whether taken orally or used topically. ‘Herbal and dietary supplement’ (HDS), ‘traditional and complementary medicine’ (T&CM), and ‘traditional, complementary and integrative medicine’ (TCIM) are retained when describing the literature that uses those terms; they are not treated as interchangeable categories. Ethnopharmacy is used here for the study and clinical interpretation of medicine use as it occurs within cultural and community contexts. Non-botanical procedures, including purely mechanical practices even when a plant object is involved, fall outside the core CTEF decision pathway and are treated only as boundary examples [10]. Related botanical and pain-mechanism evidence [11,12] should not be conflated with the mechanism of such practices.

CTEF is designed for the microclinical task that begins when a traditional or herbal exposure is disclosed during community or ambulatory pharmacy care. It does not replace comprehensive medication review, conventional interaction resources, pharmacovigilance systems, toxicology services, prescriber assessment, or local regulation. Instead, it functions as a preparation-specific translational layer within medication reconciliation: the herbal exposure is characterized in sufficient detail, interpreted against the patient’s complete regimen and clinical context, and translated into a proportionate medication-safety action.

### 1.3. Aim and Bounded Novelty Claim

This hypothesis article proposes the community-contextualized translational ethnopharmacy framework (CTEF). The term community-contextualized is used deliberately. The framework is informed by a regional community lens and by the first author’s standpoint as a clinical pharmacologist and member of the Druze community of Beit Jann, but it was not developed through formal community co-design. Local examples are drawn from a previously published contextual account [10] and are used only to generate safety questions, not as evidence of efficacy, prevalence, or community consensus.

The novelty claim is architectural and bounded rather than universal: among the comparators identified through the targeted mapping described below, no single pharmacist-facing workflow was found that explicitly combined (1) exact preparation characterization, (2) appraisal of the evidence profile separately from transferability to the patient’s preparation, (3) integration with the complete medication and clinical context, (4) consequence-sensitive action selection, and (5) culturally responsive communication and follow-up. This claim is a hypothesis about information transformation and must be independently tested rather than assumed.

## 2. Methods and Framework Development

### 2.1. Problem-Oriented Conceptual Synthesis

CTEF was developed through a problem-oriented conceptual synthesis rather than a systematic or scoping review. Five knowledge domains were integrated because each supplies a distinct part of the target decision: ethnopharmacology and traditional knowledge research; pharmacognosy, botanical quality, and regulatory monographs; herb–drug interaction, pharmacokinetic, pharmacodynamic, toxicity, and pharmacovigilance evidence; medication reconciliation and pharmacist-delivered medication management; and culturally responsive communication and health-system integration [4,5,6,7,8,13,14,15,16,17,18,19,20,21,22,23,24,25,26,27,28,29]. The design question was not whether these domains exist, but how information from them should be handed off without collapsing preparation context, evidence uncertainty, patient vulnerability, or consequence into a single binary safe/unsafe judgment.

Framework construction proceeded through four explicit operations. First, the unit of analysis was defined as the reported preparation-specific exposure rather than the botanical name alone. Second, the patient’s complete medication and clinical context was moved early in the workflow because co-medications, symptoms, organ function, frailty, pregnancy, and therapeutic-index considerations determine which mechanisms and interactions require attention. Third, evidence profile and preparation transferability were kept conceptually separate. Fourth, the workflow was required to terminate in an explicit action, communication, and follow-up output while allowing feedback when identity or exposure information is insufficient. The original seven-phase sequence was therefore consolidated into five iterative functions to reduce overlap and improve operational feasibility.

### 2.2. Literature Identification and Comparator Mapping

Literature identification was targeted and concept-driven. The pre-submission search was conducted iteratively through 8 August 2026 using PubMed/MEDLINE, publisher databases, authoritative organizational sources, reference-list checking, and citation chaining. The revision broadened comparator searching through 9 September 2026 to include terms related not only to herbal medicine and pharmacy but also to medication therapy management, evidence-to-decision frameworks, pharmacovigilance, shared decision-making, clinical toxicology, and risk-based triage. The closest retained comparators included pharmacist professional-role and T&CM integration models [4,5,7,30,31], a hospital HDS pharmaceutical-care framework [32], pharmacist medication-reconciliation and MTM models [25,33], herbal surveillance and HDI decision-support approaches [9,24,26,28,34], GRADE evidence-to-decision architecture [35], shared decision-making models [36], culturally responsive pharmacy approaches [19,20], and WHO TCIM integration guidance [21,22].

The mapping was not designed to estimate prevalence, pooled effects, or exhaustive absence. No duplicate screening, formal risk-of-bias appraisal across all included sources, or meta-analysis was performed. The original iterative search did not preserve a prospective screening log; therefore, exact historical retrieval counts cannot be reconstructed without misrepresentation. To improve auditability, Supplementary Materials provides reproducible search strings for the revision, search dates, comparator eligibility criteria, and an inventory of retained comparator families with reasons for inclusion. Selection bias and incomplete evidence capture remain possible and are treated as limitations of the distinctiveness claim.

For conceptual development, sufficiency was judged by domain coverage and comparator saturation rather than by statistical representativeness. Candidate sources had to contribute directly to one or more target functions: exposure capture, evidence appraisal, medication-context assessment, action selection, communication, documentation, or follow-up. Close counterexamples were deliberately retained even when they reduced the apparent novelty of CTEF. This approach can support a bounded conceptual hypothesis, but it cannot establish that CTEF is universally unique or superior.

### 2.3. Comparator Findings and Mechanism of Added Value

The revised mapping identified substantial overlap with existing pharmacy and decision frameworks. The proposed added value of CTEF is therefore not the existence of medication reconciliation, evidence appraisal, referral, patient-centered communication, or professional responsibility as separate practices. It is the explicit preservation of the transformation sequence between them. CTEF requires the pharmacist to specify what the patient is actually using, distinguish what the evidence says from how well it transfers to that preparation, interpret the signal against the complete regimen and patient-specific consequences, and record why a particular action was selected. The hypothesized benefit is thus inspectability and reproducibility of reasoning, not simply the collection of more categories. Table 1 summarizes the closest comparator families and the specific, bounded respect in which CTEF is distinct from each.

## 3. Proposed CTEF Architecture and Testable Hypothesis

### 3.1. Central Hypothesis, Comparators, and Failure Conditions

CTEF is a provisional decision architecture, not a scale or numerical risk score. For comparative testing, a prespecified standard medication-reconciliation comparator should include structured recording of prescription medicines, OTC products, supplements, and known traditional/herbal products plus the usual interaction resources available in the study setting but should not require preparation-level exposure capture or the explicit E/P/V/C transition logic (evidence profile, E; preparation transferability, P; vulnerability, V; consequence, C) described below. An interaction-signal-only comparator should be defined separately as a workflow in which the database or knowledge-base alert is the primary basis for triage without mandatory preparation characterization.

Central hypothesis. Compared with a prespecified standard medication-reconciliation workflow, adding structured characterization of traditional preparations and an explicit evidence–transferability–vulnerability–consequence decision sequence will improve the completeness and reproducibility of medication-safety reasoning and increase concordance with blinded external adjudication of actionability, without producing unacceptable increases in consultation burden, unnecessary escalation, or patient-reported communication harm.

The primary process outcomes should therefore be kept distinct: exposure-characterization completeness; inter-rater reproducibility; concordance with an external adjudication standard; clinical actionability; consultation time and training burden; pharmacist and patient acceptability; disclosure/documentation; and downstream medication-safety signals. Blinded adjudicators should use a prespecified external rubric based on authoritative clinical, toxicological, pharmacovigilance, and medication-safety principles rather than simply judging whether CTEF fields were completed.

Evidence against CTEF would include failure to improve exposure characterization or inter-rater agreement; no improvement in externally adjudicated actionability; excessive false-positive escalation, unnecessary referrals, or alert burden; clinically important false reassurance; unacceptable consultation time; or deterioration in patient-reported trust, respect, or willingness to disclose traditional medicine use. These failure conditions prevent process completeness from being treated as proof of clinical value.

### 3.2. Derived Testable Propositions

P1—Exposure characterization. Preparation-specific elicitation will increase the proportion of traditional/herbal exposures for which identity, preparation, amount, route, timing, duration, and intended use are sufficiently characterized for medication-safety assessment compared with standard medication histories.

P2—Reproducibility of evidence interpretation. Separating the evidence profile from preparation transferability will improve inter-rater agreement in confidence judgments and reduce unsupported reassurance or overstatement compared with unstructured or binary interaction appraisal.

P3—Action quality. Incorporating named patient-vulnerability modifiers and the consequence of missed harm will improve concordance with blinded external adjudication of actionability rather than merely increase the frequency of action changes.

P4—Disclosure without communication harm. Culturally responsive elicitation will increase disclosure and documentation of traditional preparations without increasing unsupported efficacy endorsement, patient-reported disrespect, or loss of trust.

### 3.3. Five Iterative Functions

The five functions are intentionally iterative rather than strictly linear. Function 1 characterizes the exact exposure. Function 2 reconciles the complete medication and clinical context before targeted mechanistic interpretation. Function 3 appraises the evidence profile and transferability to the reported preparation. Function 4 translates the combined information into the least intensive proportionate action using named vulnerability modifiers, consequence anchors, and explicit escalation/de-escalation rules. Function 5 communicates, documents, and follows up. If identity, dose, or preparation remains inadequate, the workflow returns to Function 1; if an active red flag or serious suspected adverse reaction is identified, urgent clinical escalation can bypass the remaining analytic sequence. Figure 1 summarizes this five-function iterative workflow, and Table 2 sets out, for each function, the minimum inputs, the resulting output, and the iteration or override trigger.

Routine use assumes a licensed pharmacist working within the local scope of practice and access to standard medication records plus authoritative drug-interaction, botanical-monograph, toxicology, and pharmacovigilance resources. Cases requiring specialist pharmacognosy, toxicology, or prescribing decisions should be referred rather than resolved within CTEF alone.

### 3.4. Evidence-to-Action Dimensions

The original A/B/C/D/U evidence classes were removed because they risked conflating study design, directness, certainty, and signal direction. E is an evidence profile rather than a score. It records (a) the source/directness of evidence—direct human, indirect human, preclinical, mechanistic/contextual, or insufficient—and (b) the direction of the safety signal—clinically important harm/interaction, evidence suggesting no clinically meaningful interaction, mixed/uncertain, or unknown. Risk of bias, precision, consistency, dose-response, and other certainty considerations should be evaluated using established evidence or causality approaches when applicable rather than being recreated within CTEF [27,35].

Before certainty, directness, or signal direction is summarized, the evidence domain should be specified as therapeutic/efficacy evidence, pharmacokinetic-interaction evidence, pharmacodynamic-interaction evidence, direct-toxicity evidence, or product-quality/contamination evidence. Evidence from these domains should not be collapsed into a single undifferentiated label.

Preparation transferability should be treated as low/unknown when botanical identity is uncertain, local and taxonomic names conflict, mixtures or chemotypes are incompletely characterized, dose is unavailable, or adulteration/contamination cannot reasonably be excluded. Low/unknown transferability prompts clarification when feasible but does not, by itself, mandate avoidance. Table 3 sets out the revised evidence-to-action dimensions, their operational anchors, and the associated decision implications.

### 3.5. Provisional Escalation, De-Escalation, and Pharmacovigilance Rules

CTEF deliberately avoids arithmetic scoring, but structured clinical judgment requires provisional transition rules. First, active severe symptoms, suspected serious toxicity, or another red flag overrides the full workflow and prompts urgent clinical assessment according to local pathways. Second, a credible interaction or toxicity signal combined with high-consequence vulnerability—for example a narrow-therapeutic-index medicine—creates an action floor of therapeutic review rather than reassurance. Third, low or unknown preparation transferability triggers clarification when feasible; it does not automatically imply avoidance. Fourth, a weak or mechanistic signal in a low-vulnerability, low-consequence context should not be escalated automatically because false alarms can cause anxiety, unnecessary discontinuation, alert fatigue, and loss of trust. Fifth, evidence suggesting no clinically meaningful interaction, when reasonably transferable to the reported preparation and accompanied by low vulnerability and consequence, can support de-escalation and proportionate reassurance. Sixth, authoritative regulatory, toxicology, interaction, or prescriber guidance supersedes CTEF when available.

Suspected adverse drug or herb-related reactions should trigger documentation of chronology, complete medication and preparation reconciliation, assessment of severity and alternative explanations, clinical escalation when indicated, and reporting through applicable pharmacovigilance systems [28]. CTEF does not introduce a new causality algorithm; it provides a structured route for making the traditional/herbal exposure visible to existing pharmacovigilance practice.

## 4. Worked Hypothetical Applications

The following cases are hypothetical demonstrations of framework logic, not validated clinical recommendations. They are included to show how the same evidence can lead to different actions when preparation transferability, co-medications, vulnerability, or consequence changes. The examples do not establish efficacy or safety of the preparations. Table 4 presents these worked hypothetical cases and the CTEF decision logic they illustrate.

### What the Worked Cases Demonstrate

Cases A and B deliberately hold the plant and preparation constant while changing the medication context. The resulting difference in action is therefore attributable to patient-specific vulnerability and consequence rather than to the botanical name itself. Case C shows the converse problem: a preclinical interaction signal should not be presented as a proven clinical interaction, yet uncertainty may still justify review when the co-medication has a narrow therapeutic index and the consequence of missed harm is high. These contrasts are the operational added-value claim that should be tested against standard medication reconciliation.

Additional botanicals discussed in the source literature illustrate boundary conditions. Olive-leaf studies provide human blood-pressure evidence, while CYP findings remain preclinical [42,43]; fenugreek trials suggest glucose-lowering effects but use heterogeneous preparations [44]; and a topical *Dittrichia viscosa*/wood-ash preparation cannot be inferred to be clinically effective from in vitro antifungal or constituent-level evidence [45,46,47]. These examples reinforce the need to keep efficacy evidence, interaction evidence, direct toxicity, product quality, and preparation transferability conceptually separate.

## 5. Practice, Cultural, and Ethical Boundaries

### 5.1. Integration with Comprehensive Medication Review

CTEF should be activated only within a complete medication review. Prescription medicines, OTC products, dietary supplements, other traditional preparations, adherence patterns, recent medication changes, and clinically relevant symptoms must be reconciled because the traditional exposure cannot be interpreted in isolation. This is consistent with pharmacist-led medication reconciliation and MTM models that emphasize medication therapy review, action planning, intervention/referral, documentation, and follow-up [25,33,48]. The framework therefore addresses a missing representation problem rather than proposing a parallel medication-review system.

Adherence support may be included in Function 5 when a medication-safety issue affects regularity, understanding, or follow-up, but adherence is not a unique pharmacological component of CTEF. Similarly, consent requirements for routine pharmacy review depend on jurisdiction, professional standards, data handling, and whether the framework is being used for care or research. Any research validation involving patient data requires the appropriate ethics and consent process.

### 5.2. Culturally Responsive Communication, Patient Agreement, and Privacy

The current framework claims culturally responsive communication, not validated cultural safety. Respectful elicitation, avoidance of ridicule, explanation of uncertainty, and separation of respect for the patient from endorsement of unsupported efficacy may improve disclosure, but cultural safety is a broader patient- and community-defined construct that includes power, institutional practice, bias, historical mistrust, and accountability [19,20,29]. Whether CTEF is experienced as culturally safe must therefore be evaluated by patients and communities rather than declared by the authors.

Awna is retained only as an optional local implementation example of trusted social support [10]. Relatives or caregivers may assist with product identification, medication recall, symptom monitoring, or continuity only with the patient’s agreement. Their involvement is never required for CTEF use and must preserve confidentiality, respect a patient’s wish not to disclose traditional practices to family members, and avoid coercion when family opinions conflict with the patient’s preferences.

### 5.3. Traditional Knowledge Provenance and Commercial Boundaries

Community-contextualized practice creates ethical obligations beyond conventional human-subject review. Reports of traditional use should be attributed to their source and should not be decontextualized into efficacy claims. Future co-design and field validation should consider consent for the use of community knowledge, appropriate acknowledgement, benefit sharing where applicable, and safeguards against commercial appropriation or misrepresentation. CTEF itself is proposed as a non-proprietary academic framework; the manuscript does not evaluate a branded herbal product, proprietary formulation, or commercial dataset.

### 5.4. International and Cross-Cultural Adaptation

The Upper Galilee examples are contextual inputs rather than the framework itself. Cross-cultural adaptation should not consist merely of replacing Galilean plants and terminology with local equivalents. Each setting will require review of local product forms, languages, regulatory sources, professional scope, referral pathways, traditional knowledge governance, and the acceptability of elicitation and communication. Local pharmacists, patients, traditional knowledge holders where appropriate, and other relevant stakeholders should participate in adaptation before claims of cultural safety or generalizability are made [21,22,29].

## 6. Staged Validation Program

Validation should proceed in stages so that operational problems are detected before patient-facing effectiveness claims are made.

Stage 1—Content validity and co-design. Independent community pharmacists, clinical pharmacists, pharmacologists/toxicologists, implementation-science expertise, patients, and community/traditional knowledge representatives should assess whether the five functions, evidence-to-action dimensions, terminology, and communication safeguards are complete, understandable, and acceptable. This stage should specifically test whether the framework is appropriately community-contextualized and identify modifications required before local implementation.

Stage 2—Manualization, simulation, and inter-rater reliability. No formal CTEF simulation study has yet been conducted. A training manual and standardized case set should be developed. Pharmacists should independently characterize exposures, record E/P/V/C, and select actions. Outcomes should include inter-rater agreement, misclassification patterns, false-positive escalation, false reassurance, failure to recognize urgent risk, and the amount of training required. Simulation is particularly important because the framework has not yet been used for direct care.

Stage 3—Feasibility and usability. Limited real-world or simulated pharmacy studies should measure completion rate, consultation time, information-resource requirements, usability, pharmacist acceptability, patient acceptability, disclosure, documentation completeness, referral burden, alert fatigue, privacy concerns, and patient-reported trust or respect.

Stage 4—Comparative evaluation. CTEF should then be compared with a prespecified standard medication-reconciliation workflow and, where relevant, interaction-signal-only screening. Blinded external adjudication should assess actionability and clinically important missed risks. Comparative studies should also measure low-value referrals or avoidance, consultation burden, pharmacovigilance capture, and patient-centered outcomes. Only after these stages should multicenter studies evaluate medication-safety outcomes and cross-cultural transportability.

A minimum dataset for future testing should include reported/local name, botanical or product identity and certainty, plant part/formulation, preparation, amount, route, frequency, timing, duration, intended use, source, complete medication list, relevant symptoms/laboratory data, evidence profile, transferability, named vulnerability modifiers, consequence category, action, communication plan, follow-up, and whether the final action agreed with independent adjudication. The proposed architecture is falsified or materially weakened if it cannot be applied reproducibly or if its process gains are offset by excessive burden or inappropriate escalation.

## 7. Limitations

CTEF remains an author-developed conceptual hypothesis. The literature synthesis and comparator mapping were targeted rather than systematic; duplicate screening, comprehensive database coverage, formal risk-of-bias assessment across all comparator sources, and quantitative synthesis were not performed. The original iterative search did not retain record counts, and a relevant overlapping architecture may therefore have been missed. The revised audit trail improves transparency but does not eliminate selection bias. Accordingly, the distinctiveness claim is restricted to the approaches identified through the stated searches and should be independently replicated.

The framework is community-contextualized rather than community co-designed. The first author’s membership in the Druze community and clinical pharmacology experience provide a relevant standpoint but cannot represent the diversity of community perspectives. Local practices derived from the contextual source [10] were not independently re-collected, prevalence estimated, or chemically verified for this article. The framework should therefore not be described as culturally safe or internationally generalizable until patient/community-defined evaluation and local co-design have occurred.

The E/P/V/C dimensions and transition rules are provisional and not validated scales. Even with anchors, reasonable pharmacists may disagree about transferability, vulnerability, consequence, and action; inter-rater reliability is an empirical question. Preparation composition may be unknown, product adulteration or contamination may be unrecognized, and evidence may be absent for the exact exposure. The workflow may also increase consultation time, referrals, false alarms, or unnecessary avoidance. These risks are explicit outcomes for future simulation and comparative testing.

Finally, CTEF addresses traditional and herbal botanical exposures within a medication review; it does not replace a conventional polypharmacy review, established drug-interaction systems, toxicology services, pharmacovigilance causality methods, or specialist judgment. It should not be used as a treatment guideline or clinical decision rule before staged validation.

## 8. Conclusions

Traditional herbal use becomes a medication-safety problem when the exposure is invisible, reduced to a plant name, or translated from uncertain evidence into either uncritical reassurance or disproportionate alarm. The proposed CTEF preserves the exact preparation, embeds it within the complete medication and clinical context, separates the evidence profile from transferability, and uses named vulnerability and consequence factors to select a proportionate action. Its scientific claim is deliberately testable: this architecture should improve characterization, reproducibility, and externally adjudicated actionability without unacceptable burden, over-escalation, or communication harm. Until co-design, simulation, reliability, feasibility, comparative, and cross-cultural studies are completed, CTEF remains a provisional framework for investigation rather than a practice-ready clinical tool.

## Figures and Tables

**Figure 1:**
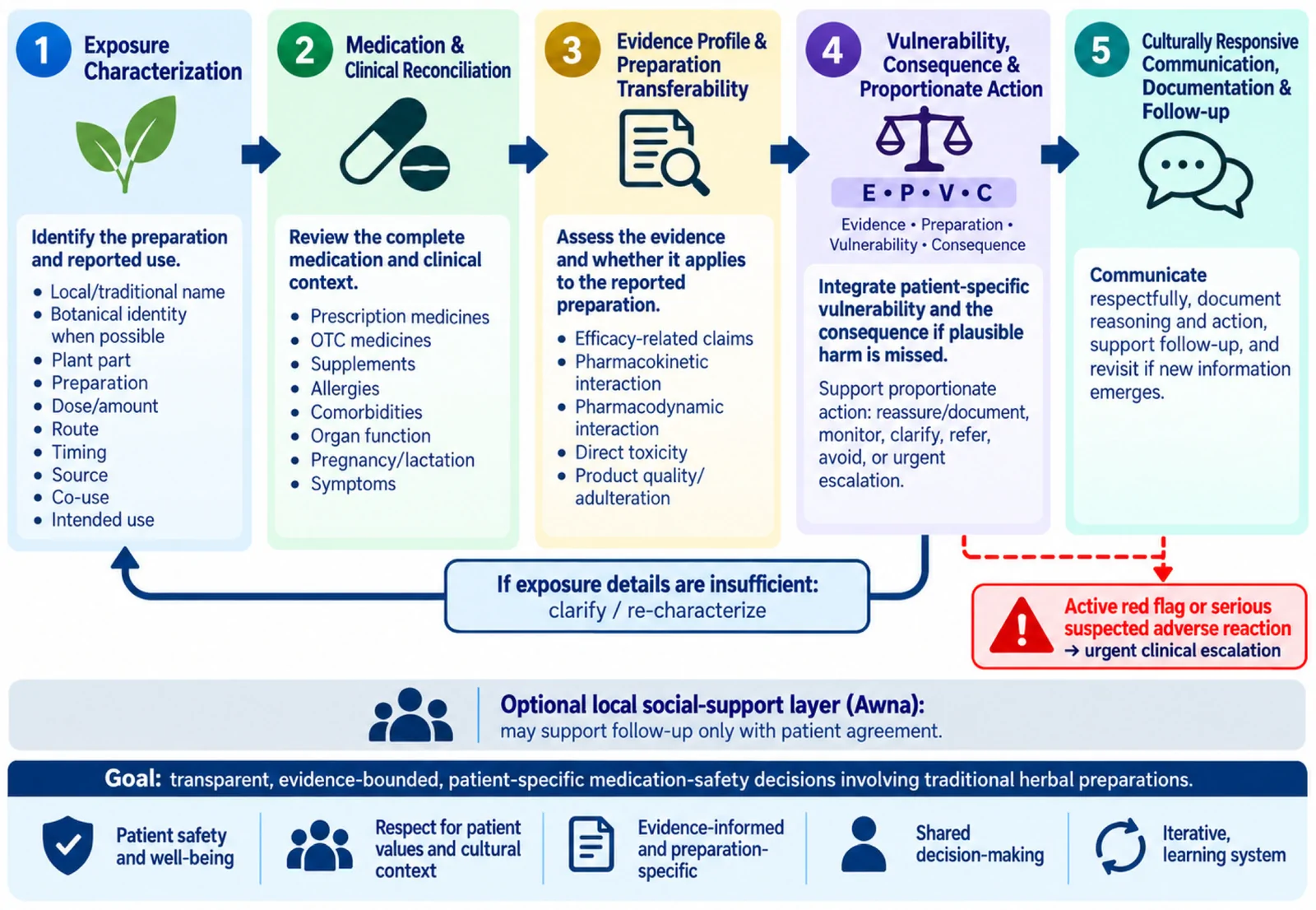
Proposed iterative community-contextualized translational ethnopharmacy framework (CTEF). The five functions integrate exact exposure characterization with complete medication reconciliation, evidence/transferability appraisal, consequence-sensitive action selection, and culturally responsive communication. Feedback is required when exposure information is insufficient, whereas active red flags or serious suspected harm trigger an urgent override. The optional Awna layer is an example of local social support and is not required for CTEF use. E, evidence profile; P, preparation transferability; V, vulnerability modifiers; C, consequence if harm is missed. Solid arrows denote the sequential progression through the five functions and the feedback return to Function 1 when exposure details are insufficient; the dashed red arrow denotes urgent clinical escalation that bypasses the remaining sequence when an active red flag or serious suspected adverse reaction is identified.

**Table 1:** Closest comparator families and bounded CTEF distinction.

Comparator Family	What It Already Contributes	Bounded CTEF Distinction
Paisansirikul & Tangkiatkumjai HDS pharmaceutical-care framework [32]	Comprehensive pharmacist responsibilities for HDS screening, product identification, interaction/herb-disease assessment, adverse effect management, counseling, documentation, and high-risk patients.	Very close service framework; CTEF adds an explicit preparation-to-evidence transferability step and consequence-sensitive transition logic that can be tested for inter-rater reproducibility.
Ung/Harnett professional-role and T&CM integration literature [4,5,7,30,31]	Defines responsibilities, competencies, counseling expectations, barriers, and strategic integration of T&CM into pharmacy practice.	CTEF operationalizes a narrower patient-level handoff from one disclosed preparation to one documented safety action.
Medication reconciliation and MTM models [25,33]	Comprehensive medication review, personal medication record, action plan, intervention/referral, documentation, and follow-up.	CTEF is not an alternative to MTM; it is a preparation-specific layer activated when a traditional/herbal exposure cannot be represented adequately by a product name alone.
Evidence-to-decision, HDI decision support, pharmacovigilance, and shared decision-making [9,24,26,28,34,35,36]	Provide structured evidence assessment, alerts/interaction guidance, adverse-reaction monitoring, risk-based recommendations, and patient–clinician deliberation.	CTEF links these functions to exact preparation transferability and community-contextualized elicitation within one pharmacist-facing microclinical workflow.

**Table 2:** Five CTEF functions, outputs, and iteration triggers.

Function	Minimum Inputs	Output	Iteration/Override Trigger
1. Characterize exposure	Reported/local name; botanical/product identity and certainty; plant part/formulation; preparation; amount; route; frequency; timing; duration; source; intended use.	Preparation-specific exposure record.	Identity, dose, product composition, or preparation remains insufficient.
2. Reconcile medication and clinical context	All prescription and OTC medicines; supplements; other traditional products; indication; symptoms/possible ADRs; relevant laboratory data; organ function; pregnancy; frailty; other risk modifiers.	Complete interaction and vulnerability context.	Active red flag or suspected serious harm triggers urgent override.
3. Appraise evidence and transferability	Evidence profile and signal direction; authoritative monographs/interaction sources; preparation transferability (P).	Documented evidence-and-transferability statement.	Uncertain identity/exposure returns to Function 1; missing clinical context returns to Function 2.
4. Translate to action	Named vulnerability modifiers (V); consequence if plausible harm is missed (C); escalation/de-escalation rules; local regulation/referral pathways.	Provisional action with explicit rationale.	Conflicting signals or specialist-level uncertainty triggers referral/deference to authoritative resources.
5. Communicate, document, follow up	Culturally responsive explanation; patient agreement/privacy; monitoring; adherence support when relevant; referral; pharmacovigilance reporting.	Documented plan and follow-up interval.	New symptoms, new medicines, or changed preparation restart the relevant function.

**Table 3:** Revised CTEF evidence-to-action dimensions and anchors.

Dimension	Operational Anchors	Question	Decision Implication
E—Evidence profile	Source/directness: direct human; indirect human; preclinical; mechanistic/contextual; insufficient. Signal direction: harm/interaction; no clinically meaningful interaction; mixed/uncertain; unknown.	What does the most relevant evidence indicate, how direct is it, and what uncertainty remains?	E describes the relationship under study; it does not by itself determine action.
P—Preparation transferability	High: identity, plant part/formulation, route, preparation, dose/amount, and timing are sufficiently comparable to the evidence source. Partial: relevant identity is known but important formulation/dose/preparation differences remain. Low/unknown: identity or exposure is poorly characterized or cannot be mapped defensibly.	How well does the evidence apply to what this patient is actually using?	Low P limits reassurance and usually prompts clarification; low P is not, by itself, evidence of high risk.
V—Vulnerability modifiers	Named modifiers rather than a global score: narrow-therapeutic-index therapy, anticoagulation, glucose-lowering therapy, pregnancy, major frailty, renal/hepatic impairment, immunosuppression, or another case-specific factor.	What patient or regimen factors increase susceptibility to harm or therapeutic failure?	V should be recorded by type; multiple modifiers can lower the threshold for review or monitoring.
C—Consequence if harm is missed	High: plausible hospitalization, irreversible injury, severe toxicity, or major therapeutic failure. Moderate: clinically meaningful but generally reversible/manageable harm. Low: limited expected clinical consequence.	What is the clinical cost of being wrong if the plausible risk is real?	Higher C lowers the threshold for escalation when a credible signal is present; low C supports proportionate de-escalation.
Action	Document/monitor; counsel; clarify product/preparation; contact prescriber or refer for therapeutic review; temporary avoidance when justified; urgent assessment for active serious harm.	What is the least intensive action consistent with the evidence, transferability, vulnerability, consequence, and authoritative guidance?	Action remains subordinate to local regulation, established interaction/toxicology resources, and specialist judgment.

**Table 4:** Worked hypothetical cases demonstrating CTEF decision logic (not clinical recommendations).

Field	Case A: Morus, Lower-Risk Context	Case B: Same Morus, Higher-Risk Context	Case C: Nigella Plus Phenytoin
Reported preparation	Morus alba leaf infusion prepared at home; one cup before the evening meal.	Same Morus alba leaf infusion and timing as Case A.	Nigella sativa oil; commercial concentrated product, dose incompletely reported.
Identity/preparation details	Leaf identified by patient photograph/local source; household infusion strength not standardized; oral use; daily for 2 weeks.	Same preparation; patient able to describe amount, timing, and source.	Product label available but thymoquinone content and exact daily exposure not established.
Intended use/relevant findings	Patient reports use for post-meal glycemic support; no relevant symptoms are reported.	Same intended use as Case A; recent low-normal home glucose readings are reported, without current severe hypoglycemic symptoms.	The intended purpose has not yet been clarified and should be elicited; no current symptoms suggesting phenytoin toxicity are reported.
Complete regimen	No glucose-lowering medicine; no narrow-therapeutic-index drug; no relevant symptoms.	Sulfonylurea plus metformin; recent low-normal home glucose readings; older adult.	Phenytoin for seizure control; no current toxicity symptoms; stable regimen.
Evidence profile (E)	Direct human evidence supports a postprandial glycemic effect for standardized 1-DNJ-enriched mulberry preparations [37,38]; evidence does not establish the effect size of this household infusion.	Same evidence profile as Case A.	Human clinical literature describes other Nigella effects [39]; thymoquinone inhibits CYP2C9 in human liver microsomes and modeling identifies a potential phenytoin interaction [40,41]. Clinical interaction magnitude remains uncertain.
Preparation transferability (P)	Partial: botanical/part/route are compatible, but dose and 1-DNJ content differ from standardized study preparations.	Partial for the same reason.	Low-to-partial: product identity is known, but active-constituent exposure and clinical transferability of the preclinical signal are uncertain.
Vulnerability (V)	No major modifier identified.	Glucose-lowering therapy and older age increase susceptibility to clinically meaningful hypoglycemia.	Narrow-therapeutic-index antiepileptic therapy is a major vulnerability modifier.
Consequence (C)	Low-to-moderate if a modest glycemic effect is missed in this context.	Moderate-to-high because additive glucose lowering could cause symptomatic hypoglycemia.	High because clinically important phenytoin overexposure or loss of seizure control can have serious consequences.
Provisional action	Document the preparation; explain uncertainty; no automatic escalation; advise symptom awareness and reassess if medicines or preparation change.	Document; counsel regarding hypoglycemia symptoms and glucose monitoring; consider pharmacist/prescriber review if use continues or readings fall; do not substitute the preparation for prescribed therapy.	Clarify dose/product; avoid unsupported reassurance; contact prescriber/therapeutic-review pathway before unsupervised continued co-use; urgent assessment if toxicity symptoms appear.
Communication/follow-up	Respectful explanation that evidence from standardized products does not establish the effect of the household infusion.	Same respectful explanation plus a patient-specific reason for greater caution despite the same plant and preparation.	Explain that the concern arises from uncertainty plus the narrow therapeutic index, not from proof that the interaction will occur; document follow-up and pharmacovigilance action if symptoms emerge.

## Data Availability

No new data were created or analyzed in this study. Data sharing is not applicable to this article. All sources supporting the conceptual synthesis and comparator mapping are cited in the manuscript; Supplementary Materials provides the comparator-mapping audit trail added for the revision.

## Supplementary Materials

The following supporting information can be downloaded at https://www.mdpi.com/article/10.3390/pharmacy14070141/s1, File S1: Comparator-Mapping Audit Trail for the Proposed Community-Contextualized Translational Ethnopharmacy Framework (CTEF). Supplementary Materials provides the targeted comparator-mapping audit trail, reproducible revision search strings, search dates, comparator eligibility criteria, and retained comparator inventory.

## Abbreviations

CTEF	Community-contextualized translational ethnopharmacy framework
CYP	Cytochrome P450
EMA	European Medicines Agency
ESCOP	European Scientific Cooperative on Phytotherapy
HDI	Herb–drug interaction
HMPC	Committee on Herbal Medicinal Products
PPAR-alpha	Peroxisome proliferator-activated receptor alpha
WHO	World Health Organization
ADR	Adverse drug reaction
MTM	Medication therapy management
OTC	Over-the-counter
TCIM	Traditional, complementary and integrative medicine
