# Supplementary material for: Translating Traditional Herbal Use into Medication-Safety Decisions: A Proposed Community-Contextualized Translational Ethnopharmacy Framework for Pharmacy Practice
Source: Pharmacy (Basel). 2026 Sep 23;14(7):141. doi: 10.3390/pharmacy14070141 (PMC13708368; doi:10.3390/pharmacy14070141)
Supplement: Supplementary file 1 [file pharmacy-14-00141-s001.zip › pharmacy-4540780-supplementary.pdf]

Supplementary Materials

Comparator-Mapping Audit Trail for the Proposed Community-Contextualized Translational Ethnopharmacy Framework (CTEF)

Manuscript: Translating Traditional Herbal Use into Medication-Safety Decisions: A Proposed Community-Contextualized Translational Ethnopharmacy Framework for Pharmacy Practice

Authors: Ziad Dabour and David H. Kinder

Revision search update: 9 September 2026

S1. Purpose and Audit Note

This appendix documents the comparator-mapping logic used to support the bounded distinctiveness claim in the revised Hypothesis article. The exercise is a targeted conceptual mapping, not a systematic or scoping review, and no PRISMA claim is made. The purpose is to make the search and comparator-selection process auditable enough for readers to evaluate whether close alternative architectures were considered.

The original pre-submission mapping was iterative and did not preserve a prospective screening log or stable record counts. Exact historical retrieval counts therefore cannot be reconstructed without creating false precision. Rather than retrospectively invent counts, the revision reports this limitation explicitly and provides reproducible search strings, search dates, eligibility criteria, and a retained-comparator inventory. The revised distinctiveness claim is correspondingly bounded to approaches identified through these searches.

S2. Information Sources and Search Dates

Source	Purpose	Date range
PubMed/MEDLINE	Concept-driven searches across herbal/T&CM pharmacy, medication reconciliation, HDI decision support, MTM, evidence-to-decision, pharmacovigilance, shared decision-making, toxicology/risk triage.	Initial iterative searching through 8 Aug 2026; broadened revision search through 9 Sep 2026.
Publisher/journal databases and article landing pages	Title/DOI and concept-term searches for close comparator papers and cited models; used to confirm article scope and functionality.	Initial search and revision verification through 9 Sep 2026.
Authoritative organizations	WHO, EMA/HMPC and other official monograph/guidance sources relevant to TCIM integration, herbal pharmacovigilance, and botanical evidence context.	Initial search through 10 Jul 2026 with focused updates through 9 Sep 2026.
Reference lists and citation trails	Backward and forward chaining from the closest pharmacist-facing papers to identify earlier responsibility models, strategic integration frameworks, reconciliation/MTM models, and adjacent decision architectures.	Performed iteratively; refreshed during revision.

S3. Reproducible Revision Search Strings

The following strings reproduce the conceptual search families used for the revision. They are intentionally broad and are not presented as a systematic-review protocol. Search interfaces change over time; title/DOI verification and citation chaining were used after retrieval of candidate comparator papers.

Search family	Reproducible concept string
A. Herbal/T&CM pharmacy frameworks	("traditional medicine" OR "complementary medicine" OR "herbal medicine" OR ethnopharmacy OR "herbal and dietary supplement") AND (pharmacist OR pharmacy OR "medication reconciliation") AND (framework OR model OR pathway OR algorithm OR "decision support")
B. Herb-drug interaction decision support	("herb-drug interaction" OR "drug-herb interaction" OR "herbal interaction") AND (screening OR surveillance OR "decision support" OR guideline OR pathway OR pharmacist)
C. Medication-management comparators	("medication therapy management" OR "medication reconciliation" OR "comprehensive medication review") AND (pharmacist OR pharmacy) AND (framework OR model OR workflow OR service)
D. Broader evidence-to-action architectures	("evidence to decision" OR "shared decision making" OR pharmacovigilance OR "clinical toxicology" OR "risk triage") AND (framework OR model OR algorithm OR pathway OR decision)
E. Cultural communication and adaptation	("culturally responsive" OR "cultural safety" OR "cultural humility" OR "ethnic minority") AND (pharmacy OR pharmacist OR medication) AND (communication OR framework OR model OR codesign)

S4. Comparator Eligibility and Selection Logic

Candidate approaches were retained when they contributed an explicit framework, model, validated tool, workflow, professional-responsibility architecture, or decision-support system relevant to one or more of the target functions: exposure capture; evidence appraisal; medication-context assessment; action selection; communication; documentation; follow-up; or adverse-reaction monitoring. Close specialty frameworks were retained when they addressed the same evidence-to-action problem even if they were not labeled ethnopharmacy.

Approaches were not considered functionally equivalent to CTEF merely because they shared individual components. The comparison focused on whether the architecture explicitly preserved the sequence from exact traditional/herbal preparation -> evidence profile separate from transferability -> complete medication/clinical context -> consequence-sensitive action -> culturally responsive communication/follow-up. The mapping was deliberately broadened to include domains that could undermine a narrow novelty claim, including MTM, evidence-to-decision, pharmacovigilance, shared decision-making, and risk-based clinical triage.

Exclusion from the closest-comparator table did not imply irrelevance. Some approaches were treated as design inputs rather than direct competitors because they operated at a different scale (e.g., health-system integration), addressed one component only (e.g., product surveillance), or lacked preparation-specific traditional/herbal exposure mapping.

S5. Retained Comparator Inventory

Comparator family	Representative source(s)	Functions already covered	Why not treated as functionally equivalent to CTEF
Hospital HDS pharmaceutical-care framework	Paisansirikul & Tangkiatkumjai 2026 [32]	HDS screening; product identification; herb-drug/herb-disease assessment; adverse-effect management; counseling; documentation; high-risk patients.	Closest service comparator. Does not explicitly separate evidence profile from transferability to the exact preparation or specify the same consequence-sensitive transition logic.
Professional-role / strategic T&CM integration	Ung, Harnett and colleagues [4,5,7,30,31]	Responsibilities, competencies, counseling, barriers, and strategic integration of T&CM in pharmacy practice.	Defines what pharmacists should do; CTEF tests a narrower patient-level transformation from one disclosed preparation to one documented action.
Medication reconciliation	AlAhmad et al. 2020 [25]	Medication-list reconciliation and pharmacist-patient-centered organization of reconciliation.	Does not inherently require household/traditional preparation characterization or separate transferability appraisal.
Medication Therapy Management	APhA/NACDS Foundation 2008 [33]	Medication therapy review; personal medication record; action plan; intervention/referral; documentation and follow-up.	Broad pharmacist service architecture. CTEF is proposed as a preparation-specific translational layer within such comprehensive review, not as a replacement.
Ethical complementary-medicine framework	Popattia & La Caze 2021 [23]	Evidence-based advice, informed recommendations, pharmacist competence, vigilance, intervention when risk is significant.	Provides ethical duties but not a preparation-specific E/P/V/C transition architecture.
Herbal-supplement surveillance tool	Jairoun et al. 2022 [24]	Product identity, labeling, storage, contraindication/use information, falsification/substandard-product surveillance.	Focuses product surveillance and safe dispensing rather than patient-specific evidence-to-action translation across uncertain household preparations.
HDI screening tools	Shakeel et al. 2020 [9]	Interaction detection across multiple screening resources.	Demonstrates value and inconsistency of interaction-screening resources; alert output is an input to CTEF rather than its endpoint.
AI/CDSS polypharmacy tool	Martins et al. 2024 [26]	Pharmacist-facing alerts and decision-support for polypharmacy/HDI knowledge.	Does not explicitly preserve culturally contextualized preparation details and separate transferability from evidence strength.
Specialty HDI decision guidance	Birdsall & Weiss 2014 [34]	Risk-based clinical decision guidance for potential HDIs in oncology.	Close evidence-to-action logic, but specialty-specific and not a general community-pharmacy workflow linking traditional preparation context, transferability, and communication.
GRADE Evidence-to-Decision	Alonso-Coello et al. 2016 [35]	Transparent movement from evidence assessment to decisions using prespecified criteria.	Strong architectural analogue at guideline/recommendation scale; not designed for one patient-reported herbal preparation in a pharmacy encounter.
Shared decision-making	Elwyn et al. 2012 [36]	Practical model for patient-clinician deliberation and preference-sensitive decisions.	Informs communication and patient partnership but does not provide pharmacognostic or preparation-transferability logic.
Herbal pharmacovigilance	WHO 2004 [28]	Safety monitoring and adverse-reaction reporting for herbal medicines within pharmacovigilance systems.	Critical follow-up/safety infrastructure; does not itself structure the full preparation-to-action pathway.
Culturally responsive pharmacy	Walpola & Harrison 2022; Robinson et al. 2022 [19,20]	Trust, access, person-centered communication, codesign recommendations for ethnic-minority pharmacy care.	Communication architecture without the pharmacological evidence/transferability kernel.
WHO TCIM integration	WHO 2025 [21,22]	Health-system policy and service-integration models; evidence, safety, regulation and responsible use.	Operates at health-system/service scale rather than the microclinical translation of one preparation in one medication context.

S6. Interpretation of the Mapping

The mapping demonstrates that the individual components of CTEF are not novel in isolation. Medication review, referral, evidence appraisal, pharmacovigilance, culturally responsive communication, and action planning are established practices. The bounded hypothesis is that explicitly preserving the handoffs among exact preparation characterization, evidence profile, transferability, vulnerability/consequence, and action may improve inspectability and reproducibility for a problem that standard systems represent incompletely.

The audit trail therefore supports only a bounded statement: within the approaches identified through the stated targeted searches, no single comparator was found that explicitly operationalized the same preparation-specific sequence in a pharmacist-facing workflow. The mapping does not establish universal uniqueness, exhaustive absence, or superiority. Those claims would require independent replication and empirical comparison.

Note: Reference numbers in this appendix correspond to the revised main manuscript.
